# Structure-Function Analyses of Human Kallikrein-related Peptidase 2 Establish the 99-Loop as Master Regulator of Activity*[Fn FN2]

**DOI:** 10.1074/jbc.M114.598201

**Published:** 2014-10-17

**Authors:** Wolfgang Skala, Daniel T. Utzschneider, Viktor Magdolen, Mekdes Debela, Shihui Guo, Charles S. Craik, Hans Brandstetter, Peter Goettig

**Affiliations:** From the ‡ Division of Structural Biology, Department of Molecular Biology, University of Salzburg, A-5020 Salzburg, Austria,; § Klinische Forschergruppe der Frauenklinik, Klinikum rechts der Isar der TU München, D-81675 Munich, Germany,; ¶ Max-Planck-Institut for Biochemistry, Proteinase Research Group, D-82152 Martinsried, Germany, and; ‖ Department of Pharmaceutical Chemistry, University of California, San Francisco, California 94143

**Keywords:** Crystallography, Enzyme Kinetics, Kallikrein, Prostate Cancer, Serine Protease, Substrate Specificity, 99-Loop, Autolytic Cleavage, Conformational Selection, Zinc Inhibition

## Abstract

Human kallikrein-related peptidase 2 (KLK2) is a tryptic serine protease predominantly expressed in prostatic tissue and secreted into prostatic fluid, a major component of seminal fluid. Most likely it activates and complements chymotryptic KLK3 (prostate-specific antigen) in cleaving seminal clotting proteins, resulting in sperm liquefaction. KLK2 belongs to the “classical” KLKs 1–3, which share an extended 99- or kallikrein loop near their non-primed substrate binding site. Here, we report the 1.9 Å crystal structures of two KLK2-small molecule inhibitor complexes. In both structures discontinuous electron density for the 99-loop indicates that this loop is largely disordered. We provide evidence that the 99-loop is responsible for two biochemical peculiarities of KLK2, *i.e.* reversible inhibition by micromolar Zn^2+^ concentrations and permanent inactivation by autocatalytic cleavage. Indeed, several 99-loop mutants of KLK2 displayed an altered susceptibility to Zn^2+^, which located the Zn^2+^ binding site at the 99-loop/active site interface. In addition, we identified an autolysis site between residues 95e and 95f in the 99-loop, whose elimination prevented the mature enzyme from limited autolysis and irreversible inactivation. An exhaustive comparison of KLK2 with related structures revealed that in the KLK family the 99-, 148-, and 220-loop exist in open and closed conformations, allowing or preventing substrate access, which extends the concept of conformational selection in trypsin-related proteases. Taken together, our novel biochemical and structural data on KLK2 identify its 99-loop as a key player in activity regulation.

## Teaser

**Background:** Serine proteases KLK2 and KLK3 clear the way for spermatozoa before impregnation.

**Results:** Enzymatic assays and structures of KLK2 elucidate its catalytic action, especially when compared with conformations of similar proteases.

**Conclusion:** Flexible loops around the active site of serine proteases open concertedly upon substrate binding.

**Significance:** This mechanistic model will stimulate the design of pharmaceutical inhibitors.

## Introduction

Human kallikrein-related peptidases (KLKs)^2^ comprise 15 serine proteases that display the chymotrypsin fold (MEROPS clan PA, family S1). The first member of this family (KLK1) was described almost a century ago (1) and subsequently named “kallikrein,” as it was detected in the pancreas, or καλλικρϵας (2). Together with KLK3 (prostate-specific antigen), whose discovery dates back to the 1960s (3), and KLK2, whose corresponding gene was isolated in the 1980s (4), KLK1 belongs to the classical kallikrein subfamily. These proteases are more closely related to each other than to the new kallikreins 4–15 (called “new” because their gradual assignment to the KLK family started at the end of the 1990s (5)); KLK1–3 share a rather special surface loop that is 11 residues longer than the corresponding 99-loop of chymotrypsin (6), and therefore, this loop is also designated the “kallikrein loop” (7).

KLK2, which was formerly called hK2 or human glandular kallikrein 1 (Uniprot identifier P20191, MEROPS entry S01.161), is relatively restricted to prostatic tissue and seminal fluid in healthy individuals (8). Current knowledge pinpoints the main physiological role of KLK2 to sperm liquefaction. On the one hand, KLK2 may activate the zymogen form of KLK3 (9, 10), although there is contradictory evidence (11). KLK3 in turn dissolves the sperm coagulum via degradation of semenogelins 1 and 2 and fibronectin (12). On the other hand, KLK2 itself is able to cleave the latter proteins at sites distinct from KLK3 (13). Maximum KLK2 activity in sperm appears immediately after ejaculation, and it decreases within 10 min due to complex formation with PCI. Because the time course of semenogelin/fibronectin degradation and loss of KLK2 *in vivo* activity coincide, it is assumed that KLK2 complements KLK3 during sperm liquefaction (14).

However, KLK2 is aberrantly expressed in a range of human malignancies (15). Hence, elevated KLK2 levels in blood may constitute a valid marker for prostate cancer either alone or in combination with levels of various KLK3 isoforms (16). Due to its narrow tissue distribution, KLK2 has been regarded as a potential drug target in prostate cancer (17) or as a prodrug activator in targeted chemotherapy (18). In prostate carcinoma, KLK2 may promote growth or metastasis of tumor cells by interacting with the urokinase-type plasminogen activator system. KLK2 is able to activate the zymogen form of urokinase-type plasminogen activator (19), which may even initiate a positive feedback loop involving further activation of pro-KLK2 by urokinase-type plasminogen activator (11). Other cancer-related KLK2 targets include plasminogen activator inhibitor-1, an inhibitor of urokinase-type plasminogen activator (20), insulin growth factor-binding proteins 2–5 (21), and protease-activated receptor 2 (22).

Although KLK1 (23), KLK3 (24, 25), and several new kallikreins (for review see Ref. 26) are well characterized on the structural level, the structure of KLK2 has remained elusive. To close this knowledge gap within the classical kallikreins, we present here two crystal structures of KLK2 obtained from *Escherichia coli* expression and refolding. Furthermore, we characterized a series of KLK2 mutants to elucidate its Zn^2+^ inhibition and inactivation by proteolytic cleavage within the 99-loop. Kinetic properties of these mutants extend an in-depth comparison of KLK2 with related structures and investigate the diverse roles of the 99-loop in the regulation of KLK2 activity.

## EXPERIMENTAL PROCEDURES

### 

#### 

##### Cloning, Protein Expression, and Refolding

First, KLK2 expression vectors were prepared from prostate adenoma cDNA by inserting the sequence of the mature protease (Ile-16 to Pro-245a) between the BamHI and HindIII sites of the pQE-30 plasmid (Qiagen, Hilden, Germany). Hence, the resulting plasmid pQE-30-pro(DDDDK)_KLK2 encoded an N-terminal artificial propeptide (MRGSHHHHHHGSDDDDK) with a hexahistidine tag preceding the canonical enterokinase (EK) recognition sequence (DDDDK). Second, round-the-horn site-directed mutagenesis^3^ was employed to generate two vectors with alternative cleavage sites in the propeptide: pQE-30-pro(SGDR)_KLK2 and pQE-30-pro(PSFR)_KLK2. Third, we generated six point mutants from these three pQE-30 derivatives by round-the-horn site-directed mutagenesis: H25A, H91A, K95eM, K95eQ, H95fA, H101A. DNA sequencing confirmed the correctness of all constructs. Enzymes for cloning were purchased from Thermo Scientific (Waltham, MA) or Stratagene (La Jolla, CA).

KLK2 was expressed as inclusion bodies and folded *in vitro* essentially as described for the catalytic domain of EK (28). In brief, *E. coli* M15[pREP4] cells (Qiagen) were transformed with the respective expression plasmid and grown in LB medium (supplemented with 100 μg/ml ampicillin and 30 μg/ml kanamycin) until the culture reached an *A*_600_ of 1.2. Protein expression was induced with 0.5 mm isopropyl β-d-1-thiogalactopyranoside for 4 h at 37 °C. Cells were disrupted by sonication, and insoluble matter was washed with Triton X-100- and EDTA-containing buffers. Washed inclusion bodies were solubilized 1:20 (w/v) in 7.5 m guanidine-HCl, pH 9, 50 mm Tris, 100 mm β-mercaptoethanol for 24 h, dialyzed against 5 mm citrate, pH 3.5–4.0, and resolubilized 1:10 (w/v) in 7.5 m guanidine-HCl, pH 4.0–4.5, 50 mm Tris for several hours. Dropwise dilution of this solution into the 100-fold volume of 500 mm arginine, 50 mm Tris, pH 8.3, 20 mm NaCl, 1 mm EDTA, 5 mm cysteine-HCl, and 0.5 mm cystine yielded 5–10% folded protein after 3 days at 16 °C.

##### Protein Purification

**FIGURE 1.**
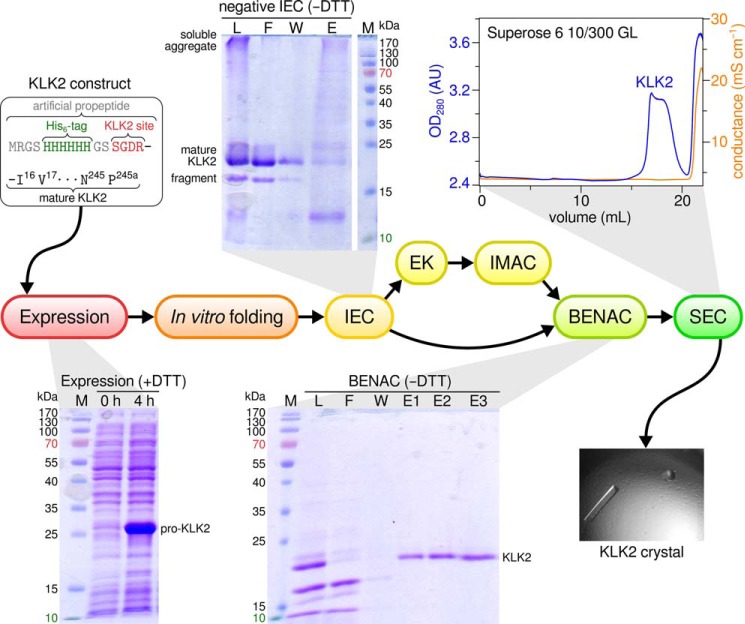
**Purification scheme for recombinant KLK2.** Purification comprised negative IEC, activation of pro-KLK2 by EK, immobilized metal ion affinity chromatography (*IMAC*), BENAC, and size exclusion chromatography (*SEC*). Success of the individual steps was routinely monitored by SDS-PAGE. +*DTT* and −*DTT* indicates whether gels were run under reducing or oxidizing conditions, respectively. *M*, marker; *L*, load; *F*, flow-through; *W*, wash; *E*, elution; *AU*, absorbance units.

Wild type KLK2 and the mutants K95eM, K95eQ, and H101A were purified by (negative) ion exchange chromatography (IEC) and benzamidine (BEN) affinity chromatography (BENAC); purification of the mutants H25A, H91A, and H95fA comprised (positive) IEC, activation by EK, negative metal ion affinity chromatography, and BENAC (Fig. 1). Chromatography resins were obtained from GE Healthcare. After tangential flow concentration, the refolding solution was loaded onto a Q-Sepharose column equilibrated in IEC buffer (50 mm Tris-HCl, pH 8.0). The ratio of load to resin was about 50:1 (v/v) in this and all following affinity chromatography steps; all buffers contained 3 mm sodium azide. In negative IEC, the flow-through contained mature KLK2. In positive IEC, pro-KLK2 was eluted from the column with 3 resin volumes of IEC buffer supplemented with 150 mm NaCl.

Zymogen forms of KLK2 from positive IEC were incubated with EK in a molar ratio of 1000:1 for 15 h at 20 °C. EK was produced in-house as previously described (28). The digestion mixture was brought to 500 mm NaCl, 10 mm imidazole and loaded onto a Ni^2+^- or Co^2+^-Sepharose column equilibrated in 50 mm Tris-HCl, pH 8.0, 500 mm NaCl, 10 mm imidazole. The flow-through contained mature KLK2, whereas the resin-bound residual pro-KLK2 cleaved propeptide and EK.

For BENAC, flow-through from negative IEC was brought to 500 mm NaCl and loaded onto a benzamidine-Sepharose column equilibrated in BENAC buffer (50 mm Tris-HCl, pH 8.0, 500 mm NaCl). Flow-through from metal ion affinity chromatography was directly loaded due to its proper sodium chloride concentration. After washing with 8 resin volumes of BENAC buffer, bound KLK2 was eluted with 3 × 2.5 resin volumes of BENAC buffer supplemented with 25, 50, and 100 mm benzamidine.

As final polishing step, size exclusion chromatography was performed over a Superose 6 10/300 GL column connected to an ÄKTA FPLC system (GE Healthcare). To this end, the BENAC eluate was concentrated in Amicon Ultra-15 Centrifugal Filter Units, molecular weight cutoff 10 kDa (Millipore, Billerica, MA). Per run, 500 μl of concentrate were loaded onto the column at 4 °C (running buffer: 20 mm Tris-HCl, pH 8.0, 20 mm NaCl, 5 mm benzamidine). Fractions that corresponded to the monomeric KLK2 peak were combined and concentrated to 12 mg/ml. Chemicals of the highest purity available were either from AppliChem (Darmstadt, Germany), Carl Roth (Karlsruhe, Germany), Merck, or Sigma.

##### Enzyme Kinetics and Inhibitory Studies

Bz-PFR-pNA, H-GHR-AMC, H-PFR-AMC, H-Arg-AMC, and PPACK were obtained from Bachem (Weil am Rhein, Germany). Amidolytic activity was generally measured in 100 μl of assay buffer (50 mm Tris-HCl, pH 7.5, 100 mm NaCl, 10% (v/v) DMSO, 0.1% (w/v) BSA) containing 400 ng (150 nm) of KLK2 and 250 μm chromogenic or fluorogenic substrate. pH values of the reaction mixtures were routinely checked to exclude any effects of pH changes. Time-dependent substrate cleavage corresponded to changes in absorbance at 405 nm (for pNA substrates) or fluorescence at 460 nm (for AMC substrates; excitation wavelength: 380 nm) and was recorded on an Infinite M200 microplate reader (Tecan, Männedorf, Switzerland). Protein concentrations were determined by absorbance at 280 nm using computed extinction coefficients and molecular weights. For calculating *k*_cat_ values, we performed active site titration of the respective KLK2 variant with PPACK and corrected the data accordingly.

The pH optimum was determined in 100 mm SPG buffer (12.5 mm succinate, 43.75 mm NaH_2_PO_4_, 43.75 mm glycine). Zn^2+^ inhibition curves were measured without BSA, as its metal binding sites sequestered Zn^2+^ ions from the reaction buffer. However, KLK2 adsorbed to the microplate walls in the absence of BSA, which interfered with the measurement of Michaelis-Menten kinetics at different Zn^2+^ concentrations. Thus, we saturated all Zn^2+^ binding sites in BSA by dialyzing BSA-containing assay buffer against the 200-fold volume of assay buffer with the desired Zn^2+^ concentration. Reactivity toward the burst reagent 4-nitrophenyl-4-guanidinobenzoate (NPGB) (29) was determined by adding 75 μl of 100 μm KLK2 to 675 μl of 50 mm HEPES, pH 7.0, 150 mm NaCl, 50 μm NPGB and by detecting the concomitant change in absorbance at 405 nm. Substrate specificity was determined by positional scanning as previously described (30). Data were analyzed with nonlinear regression models as implemented in QtiPlot v0.9.8.8 (31).

##### Crystallization and Data Collection

Crystals of active wild type KLK2 were grown at 20 °C by vapor diffusion in 500 nl of sitting drops of a 12 mg/ml protein solution in 20 mm Tris-HCl, pH 8.0, 20 mm NaCl, 10 mm benzamidine, 3 mm NaN_3_ that were mixed with 500 nl of precipitant. Crystals of benzamidine-inhibited KLK2 appeared within 10 days in 500 mm (NH_4_)_2_SO_4_, 1 m Li_2_SO_4_, 100 mm sodium citrate and were directly frozen in the nitrogen gas stream (100 K) without prior cryoprotection. Crystals of the KLK2-PPACK complex were obtained by soaking KLK2-benzamidine crystals for 1 h in mother liquor supplemented with 7 mm PPACK. Data sets were collected in-house (Bruker AXS microstar rotating anode, mar345 image plate) or at the beamline X12 (MarMosaic 225 charge-coupled device) at the DESY in Hamburg (see Table 1).

##### Structure Determination and Refinement

Diffraction data were integrated by iMosflm v1.0.5 (32) and scaled with Scala v3.3.20 (33) included in the CCP4 v6.2.0 program suite (34). Initial phases were obtained for the KLK2-BEN data set by molecular replacement with Phaser v2.3.0 (35) using KLK3 (2zch/chain P) as the search model in the resolution range of 1.9–36.0 Å. The scores of the top solution were RFZ = 17.5, TFZ = 32.9, LLG = +1456, and R-factor = 45.8. Neither the rotational nor the translational searches yielded a second unrelated peak. Phases for the KLK2-PPACK data were obtained by molecular replacement using Phaser with the KLK2-BEN polypeptide model after refinement. Essentially, the parameters were similar to the initial search, resulting in an LLG = +2688 and an R-factor = 34.8. Both KLK2 structures contained one molecule in the asymmetric unit and had a Matthews coefficient of 2.33 and solvent content of 47.2%. Model building in Coot v0.6.2 (36) alternated with restrained maximum likelihood refinement in *REFMAC* v5.6.0117 (37) using standard target parameters (38). Global quality indicators of the final models were in the typical range for the obtained resolution (see Table 1). Geometric restraints for the two covalent bonds between PPACK and KLK2 were determined in JLigand v1.0.36 (39); two CIF files were generated, each describing one bond, and then manually merged (supplemental File S1). Correct Asn and Gln side chain rotamers were assigned by NQ-Flipper v2.7 (40). Side chains of the following surface residues lacked interpretable electron density in both models unless otherwise indicated: Lys-24, Lys-60 (only KLK2-BEN), Lys-61, Pro-76, Arg-82 (KLK2-BEN), His-87, Glu-110, Lys-113, Asp-116 (KLK2-PPACK), Lys-119, Asn-128, Glu-129 (KLK2-BEN), Glu-148, Arg-153, Glu-174 (KLK2-BEN), Lys-175, Glu-178 (KLK2-BEN), Glu-218 (KLK2-BEN), Arg-235 (KLK2-BEN), Lys-236, Lys-239 and Pro-245a (KLK2-BEN). As recommended (41), these side chains were modeled in their most likely conformation with full occupancy, which resulted in high B values. Met-167-Sδ was modeled with two alternate conformations in the side chain beyond Cγ. Structures were validated by MolProbity v3.19 (42), *SFCHECK* v7.03.16 (43) and phenix.model_vs_data (44), included in the Phenix v1.7.3–928 suite of programs (45).

##### In Silico Analyses and Data Visualization

KLK2-BEN and KLK2-PPACK were compared with the following structures (PDB IDs are in parentheses): the classical human kallikreins KLK1 (1spj) (23) and KLK3 (2zch, 3qum) (24, 25); the new human kallikreins KLK4 (2bdh) (46), KLK5 (2psx) (47), KLK6 (1l2e, 3vfe) (48, 49), pro-KLK6 (1gvl) (50), KLK7 (2qxg, 3bsq) (51, 52), and KLK8^4^; porcine pKLK1 (2pka, 2kai, 1hia) (7, 53, 54); rat rKlk1c2 (1ton) (55); equine eKLK3 (1gvz) (56); mouse mKlk1b4 (1sgf) (57) mKlk1b26 (1ao5) (58), and mKlk8 (1npm) (59); bovine trypsin (1ce5) (60) and chymotrypsin A (1yph).^5^ The chymotrypsinogen numbering scheme of KLK2 was derived from a structure-based sequence alignment to chymotrypsin, which was calculated by STRAP v2013.09.16 (62).

Dendrograms that illustrate relationships between loop conformations in the KLK family were prepared as follows. Protease structures were aligned in PyMOL by the CEalign package (63). A PyMOL script (supplemental File S2) calculated mutual dissimilarities for a target loop and stored them in a square distance matrix (we define the mutual dissimilarity of a certain loop in two superposed structures as the r.m.s.d. between loop Cα atoms with equal residue numbers in these structures). From this distance matrix the NEIGHBOR program in the PHYLIP v3.695 package (64) constructed a dendrogram according to the UPGMA algorithm.

Electrostatic surface potentials were evaluated with APBS v1.4 (65). Average B values over main- and side-chain atoms were calculated by Baverage v6.2. Benzamidine binding sites were scored by DSX v0.8.9 (66). Electron density maps were created by fft v6.2 (67). Transitions between Zn^2+^-free and Zn^2+^-bound KLK2 models were generated by the Yale Morph2 server (68). Interfaces between symmetry-related protein molecules were determined by the PISA server v1.48 at the European Bioinformatics Institute (69). Secondary structure was assigned by DSSP v2.2.1 (70). Furthermore, the following programs were used for data visualization: TeXShade v1.24 (71) for sequence alignments, TreeGraph v2.0.50 (72) for dendrograms, WebLogo v2.8.2 (73) for residue frequencies at cleavage sites, and PyMOL v1.5.0.1 (74) for protein structures.

## RESULTS

### 

#### 

##### Preparation of Wild Type and Mutant KLK2

A zymogen form of wild type KLK2, whose propeptide contained the canonical EK recognition site (DDDDK), suffered from unspecific fragmentation during activation by EK, as already observed by Lövgren *et al.* (75). Because we expected these cuts to decrease protein homogeneity, we designed two alternative propeptides that differed in their recognition site; on the one hand, the sequence SGDR most likely constituted a more efficient EK site (76) and, therefore, would require significantly smaller amounts of EK, resulting in less unspecific cuts. On the other hand, we deduced from KLK2 specificity profiling (see below) that the sequence PSFR represented an autoactivation site and would eliminate the need for EK in the first place. Interestingly, both sequences ensured that autoactivation of refolded pro-KLK2 proceeded to completion within 48 h. Accordingly, we selected the SGDR propeptide for all expressions of the wild type peptidase, as it doubled refolding yields compared with the PSFR propeptide. Final yields of wild type KLK2 exceeded 10 mg per liter of bacterial culture. The protein was >95% pure and monomeric, as judged from SDS-PAGE and size exclusion chromatography (Fig. 1).

##### Proteolytic Activity and Specificity

**FIGURE 2.**
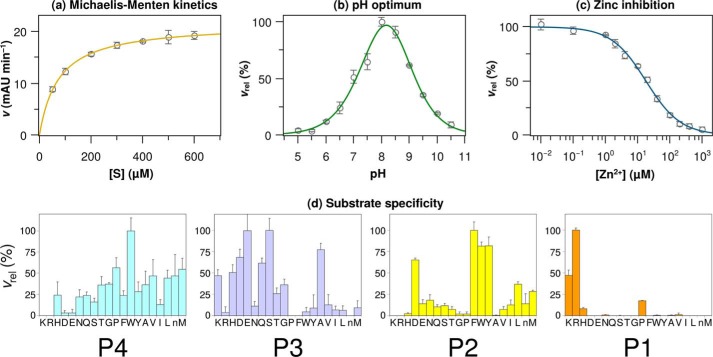
**KLK2 kinetics and substrate specificity.** *a–c*, all measurements involved 150 nm wild type KLK2 and 250 μm Bz-PFR-pNA. The pH optimum was determined in 100 mm SPG buffer. Relative velocities *v*_rel_ are relative to the highest velocity in the respective panel. *d*, substrate specificity of KLK2 as determined by a diverse positional scanning library (30). The *y* axis represents the substrate cleavage rate relative to the highest rate observed for this position. The *x* axis indicates the amino acid held constant at each position (*n*, norleucine).

Refolded KLK2 cleaved the chromogenic substrate Bz-PFR-pNA following Michaelis-Menten kinetics with *K_m_* and *k*_cat_ values of 75 ± 2 μm and 1.23 ± 0.02 s^−1^, respectively (Fig. 2*A*, see Table 2). A pH value around 8 was optimal for its proteolytic activity (Fig. 2*B*). The fluorogenic H-PFR-AMC turned out to be the best substrate for KLK2 so far, resulting in *K_m_* and *k*_cat_ values of 69 ± 3 μm and 23.42 ± 0.09 s^−1^. Lövgren *et al.* (13) reported a comparable pH optimum and *K_m_* and *k*_cat_ values of 40 μm and 0.92 s^−1^, respectively, for the cleavage of H-PFR-AMC by KLK2 expressed in eukaryotic cells. The lower *K_m_* value for the latter substrate may result from favorable interactions between its fluorogenic AMC group and the S1′ site. By contrast, the significantly higher turnover number of *E. coli* KLK2 for H-PFR-AMC might depend on a more accessible active site. A glycosylated Asn-95 in KLK2 from eukaryotic expression could favor a closed 99-loop conformation and reduced turnover. Zn^2+^-inhibited KLK2 with an IC_50_ value of 22 ± 1 μm and a *K_i_* of 4.9 ± 0.4 μm (Fig. 2*C*), which is similar to the values found by Lövgren *et al.* (13).

Positional scanning with fluorogenic peptide substrates from a synthetic combinatorial library (30) confirmed that KLK2 is a protease with predominantly tryptic specificity (Fig. 2*D*). Accordingly, P1 residues (applying the Schechter and Berger (77) nomenclature) are restricted to basic side chains, with Arg preferred ∼2-fold over Lys and only a minor appearance of His. Surprisingly, KLK2 accepts proline as third best residue in P1. The S2 pocket strongly favors the aromatic residues Phe, Tyr, and Trp, whereas Asp is similarly preferred; Leu and Met are also allowed. By contrast, basic (Lys, Arg, His) and small hydrophobic side chains (Gly, Ala, but also Pro) are nearly excluded in P2. Substrate specificity is less pronounced for the S3 and S4 sites. In accordance with the solvent-exposed S3 subsite, charged, polar, and small side chains are accepted (Ser, Glu, Ala, Asp, Gln, Lys) with the notable exception of Arg, whereas all hydrophobic residues are hardly tolerated at this position. In contrast, S4 accepts hydrophobic and to a lesser extent polar side chains, with Trp being strongly preferred over Pro, Met, and several other residues.

##### Crystallization and Overall Structure

One batch of purified KLK2 yielded single crystals of the benzamidine-inhibited peptidase (KLK2-BEN) that diffracted in-house up to 1.9 Å. These crystals could also be soaked with PPACK (KLK2-PPACK), which left the unit cell parameters essentially unchanged (see Table 1). Only two interface areas between symmetry-related protein molecules exceed 400 Å^2^ (707 Å^2^ and 533 Å^2^, respectively). Moreover, the PISA server classified these interfaces as a result of crystal packing. In agreement with these findings, size exclusion chromatography and dynamic light scattering detected KLK2 monomers exclusively independent of refolding protocol and buffer conditions.

**TABLE 1:** **Data collection and refinement statistics for KLK2** Quality indicators are reported as defined by Einspahr and Weiss (105). *R*_rim_ and *R*_pim_ are the redundancy independent R-factor and the precision indicating R-factor, respectively.

	Structure	
	KLK2-BEN	KLK2-PPACK
PDB ID	4nfe	4nff

**Data collection**		
Wavelength (Å)	1.5418	0.97004
Space group	*P*2_1_2_1_2_1_	*P*2_1_2_1_2_1_
Cell constants (Å)	*a* = 59.65, *b* = 60.39, *c* = 67.68	*a* = 60.10, *b* = 60.74, *c* = 66.80
	α = β = γ = 90°	α = β = γ = 90°
Resolution range*^a^* (Å)	45.06-1.90 (2.00-1.90)	30.37-1.90 (2.00-1.90)
Number of observations*^a^*	127,875 (16,104)	122,496 (10,451)
Number of unique reflections*^a^*	19,455 (2,731)	19,421 (2,619)
Multiplicity*^a^*	6.6 (5.9)	6.3 (4.0)
Completeness*^a^* (%)	98.1 (96.5)	97.9 (92.4)
Mean 〈 *I*/σ(*I*)〉*^a^*	13.0 (5.7)	10.0 (2.3)
*R*_merge_*^a^*	0.088 (0.224)	0.150 (0.585)
*R*_rim_*^a^*	0.095 (0.247)	0.163 (0.666)
*R*_pim_*^a^*	0.036 (0.099)	0.062 (0.306)
*B* factor from Wilson plot (Å^2^)	16.5	14.2

**Refinement**		
Resolution range*^a^* (Å)	29.83-1.90 (1.95-1.90)	29.27-1.90 (1.95-1.90)
Completeness*^a^* (%)	97.8 (95.6)	97.7 (90.9)
Reflections used in refinement*^a.b^*	19,418 (1272)	19,387 (1201)
Reflections in working set*^a^*	18,433 (1200)	18,399 (1145)
Reflections in test set*^a^*	985 (72)	988 (56)
*R*_cryst_ (%)*^a^*	17.7 (18.8)	19.7 (25.2)
*R*_free_ (%)*^a^*	21.3 (27.5)	23.2 (31.4)
Residues refined	227	229
Non-hydrogen protein atoms*^c^*	1,754 (20.1)	1,771 (14.0)
Non-hydrogen ligand atoms*^c^*	33 (29.0)	30 (14.6)
Solvent water molecules*^c^*	136 (26.8)	100 (17.2)
r.m.s.d. bond lengths (Å)	0.012	0.014
r.m.s.d. bond angles (°)	1.473	1.770

**Ramachandran plot*^d^***		
Favored regions	97.3% (217/223)	98.2% (221/225)
Allowed regions	2.7% (6/223)	1.8% (4/225)
Disallowed regions	0% (0/223)	0% (0/225)

*^a^* Values in parentheses are for the highest resolution shell.

*^b^* Cutoff criterion *F* > 0 σ*_F_*.

*^c^* Average *B* values (Å^2^) in parentheses.

*^d^* Regions as defined by MolProbity (42).

**FIGURE 3.**
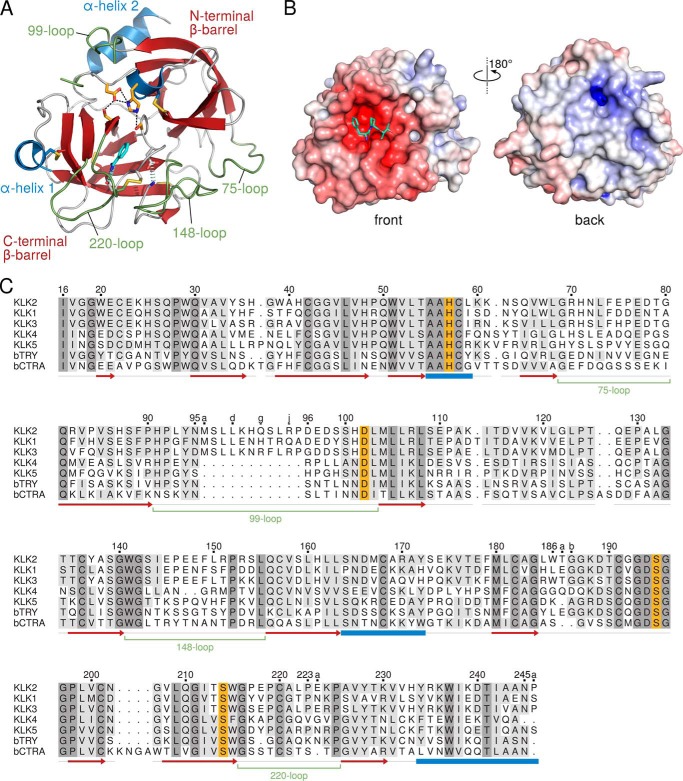
**Primary, secondary, and tertiary structure of KLK2.** *A*, overall structure of KLK2 in complex with benzamidine (*cyan*), shown in standard orientation (79), in which the substrates bind to non-primed subsites from the *left* and to primed subsites to the *right of the scissile bond*. Helices (*blue*), β-strands (*red*), and the 75-, 99-, 148-, and 220-loop (*green*) are shown as *ribbons*. Disulfide bridges (*yellow*), the active site residues Ser-214, Asp-102, His-57, and Ser-195 (*orange*), and the stabilizing salt bridge Ile-16–Asp-194 and Asp-189 at the *bottom of the S1 pocket* (*gray*) are drawn as *sticks. B*, molecular surface of KLK2 bound to PPACK. Colors relate to the electrostatic surface potential (−10 to +10 *k*_B_*T*), which was calculated by APBS (104). Note the negatively charged (*red*) substrate binding site at the front of the molecule and the positively charged (*blue*) surface next to the C-terminal α-helix at the back. *C*, structure-based amino acid sequence alignment of KLKs 1–5, bovine trypsin, and bovine chymotrypsin A. Residue numbers according to the chymotrypsinogen numbering scheme appear on *top*. The secondary structure of KLK2 is indicated *below each row*. A *light* or *dark gray background* denotes residues that are similar in at least four sequences or completely conserved, respectively. The remaining colors correspond to *A*. Minor deletions with respect to chymotrypsin A (*bCTRA*) are found in surface loops after His-36 and Lys-61, Thr-125, and Asn-202 as well as minor insertions after Trp-186, Pro-223, and the C-terminal Asn-245. The 11-residue insertion following Asn-95 is known as extended 99- or kallikrein loop of the classical KLKs 1–3. The alignment was prepared with STRAP (62) and visualized with TeXshade (71).

The KLK2 molecule resembles an oblate ellipsoid with diameters of 35 and 50 Å, respectively (Fig. 3, *A* and *B*). In addition to the two six-stranded β-barrels of trypsin-like serine proteases, residues 20–21 form a short β-strand adjacent to strand B of the C-terminal barrel (chymotrypsinogen numbering scheme) (78) adapted for kallikreins (7). In KLK2-PPACK, a 3_10_-helix comprises residues 95–95d of the 99-loop. KLK2 contains five disulfide bonds and the typical *cis*-Pro-219 observed in nearly all other kallikrein structures. Due to the prokaryotic expression system, both KLK2 structures lack any glycosylation.

In terms of Cα r.m.s.d., KLK2 is most similar to the classical KLKs (KLK3, 0.97 Å; KLK1, 1.06 Å) followed by KLK8 (1.24 Å), KLK7 (1.38 Å), KLK5 (1.40 Å), KLK6 (1.72 Å), and KLK4 (1.83 Å). Moreover, KLK2 superposes remarkably well with trypsin (1.15 Å) and chymotrypsin (2.02 Å), which are compared in a structure-based sequence alignment (Fig. 3*C*). The benzamidine- and PPACK-containing KLK2 structures superpose with a Cα r.m.s.d. of 0.88 Å, which is mainly explained by backbone deviations around the 99-loop, Glu-174, and Trp-215 in KLK2-PPACK.

Overall, the substrate binding site of KLK2 has a slightly negative potential, which is prominent at the S1 pocket (Fig. 3*B*). Two positive surface potential regions exist: first, at the back of the 37-loop with Trp-67 and Arg-82; second, at His-48 and the C-terminal Arg-235, Lys-236, and Lys-239. This extended, positively charged patch at the back of the protein corresponds partially to the anion binding exosite II of thrombin (79). It may bind allosteric effectors like heparin, which accelerates the association of KLK2 and PCI 4-fold (13).

##### Active Site Cleft

Adjacent to the extended catalytic triad (Asp-102, His-57, Ser-195, and Ser-214), the backbone amide NH groups of Gly-193 and Ser-195 constitute the oxyanion hole, which is occupied by a sulfate ion in the KLK2-BEN complex structure. The amidine group of benzamidine (BEN 301) and the carboxylate group of Asp-189 at the bottom of the S1 pocket interact via a symmetrically bidentate salt bridge. BEN-N2, which points toward the surface of the protein, also forms a hydrogen bond to the amide oxygen of Pro-217, similarly as in the KLK6-BEN complex (48). Also, BEN-N1, which points toward the core of the protein, binds to Thr-190-Oγ1, Tyr-228-Oη, a water molecule (w412), and Asp-189-Oδ1, sitting at the corners of a pentagonal hydrogen bond network. The aromatic ring of BEN lies between the peptide bonds Trp-215–Gly-216 and Cys-191–Gly-192.

Thr-190 explains why KLK2 prefers Arg merely 2-fold over Lys, in contrast to other trypsin-like peptidases (80); its Oγ atom provides a hydrogen bonding partner for P1-Arg-Nη. Consequently, KLK2 binds P1-Arg substrates too tight for efficient catalysis. Ala-226 might further decrease the preference for Arg in P1, as its methyl side chain is in close contact with the P1-Arg guanidinium group (81).

**FIGURE 4.**
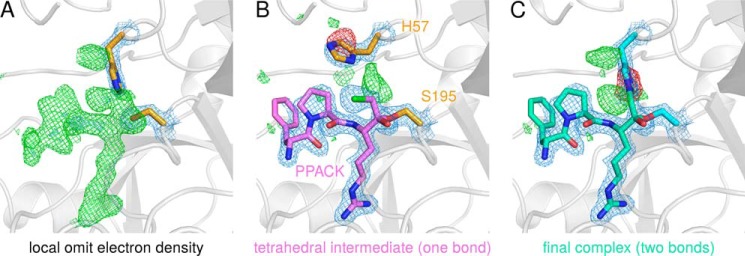
**Different KLK2-PPACK complexes that form during the inhibition reaction.** *A*, positive difference electron density (*green*), which was generated by refining KLK2-PPACK data in the absence of the ligand, indicates missing atoms in the substrate binding site. *B*, model of the tetrahedral intermediate of the inhibition reaction. This intermediate forms one covalent bond to Ser-195 (0G6-C2 to Ser-195-Oγ); the chlorine atom of the chloromethyl ketone group is still present. The tetrahedral intermediate is present in the crystal with low occupancy. *C*, the final KLK2-PPACK complex, which is characterized by a second covalent bond between the inhibitor and its target (0G6-C3 to His-57-Nϵ2). Note that the deposited KLK2-PPACK coordinates (PDB ID 4nff) model the latter situation only. Maximum likelihood-based difference electron densities are drawn in *blue* (2*mF*_obs_ − *DF*_calc_ map contoured at 1.5σ), *green*, and *red* (*mF*_obs_ − *DF*_calc_ map contoured at +3σ and -3σ, respectively). Structure factors and electron densities were calculated by REFMAC (37) and fft (67), respectively. Because crystallographic programs do not contain parameters for the two covalent bonds between PPACK and KLK2, they were generated with the software JLigand (39) and written to a crystallographic information file (CIF), which was employed in crystallographic refinement (supplemental File S1).

To avoid bias regarding the existence of PPACK in the KLK2-PPACK model, we also calculated electron density in the absence of the ligand (82). This approach yielded well defined positive difference electron density in the nonprimed substrate binding sites, which we could clearly identify as PPACK (Fig. 4*A*). However, electron density around His-57 indicated that the KLK2-PPACK crystal consisted of two species of protease-inhibitor complexes. One species contained only one covalent bond between PPACK (0G6-C2) and Ser-195-Oγ, characteristic for the tetrahedral epoxy ether intermediate of the chloromethyl ketone inhibition reaction (83). To avoid clashes with the chlorine atom, His-57 bends away from Ser-195 in this intermediate complex (Fig. 4*B*). The other species contained two covalent bonds between PPACK and KLK2 (0G6-C2 to Ser-195-Oγ and 0G6-C3 to His-57-Nϵ2) and, therefore, represents the final protease-inhibitor complex (Fig. 4*C*). Despite the heterogeneous composition of the crystal, the deposited KLK2-PPACK structure represents the covalent adduct with both His-57 and Ser-195. We believe that the short soaking time (30 min) and the initial presence of competing benzamidine in the S1 site delayed the reaction of KLK2 with PPACK.

Backbone atoms of PPACK and KLK2 form a set of five “canonical” hydrogen bonds (84) in the KLK2-PPACK structure. On the one hand, two hydrogen bonds connect P1-Arg-O to the oxyanion hole, and one hydrogen bond links P1-Arg-N and Ser214-O. On the other hand, P3-d-Phe forms the typical antiparallel β-sheet with Gly-216, serving as the S3 site. The strongest side chain interaction is made by the guanidinium group of P1-Arg with the same atoms as benzamidine in KLK2-BEN. In addition, three ordered water molecules bridge the gap between P1-Arg-Nϵ and the carboxyl group of Glu-218, which is significantly stabilized. By contrast, Glu-218 lacks electron density from Cγ on in KLK2-BEN. The P2-Pro hardly interacts with the S2 side chains of His-57, Tyr-94, Leu-95c, Ser-99 (top) and Trp-215 (back). Due to the d-configuration of P3-d-Phe, its side chain fully occupies the S4 site, where it participates in T-shaped π-stacking with Trp-215 and contacts Glu-97, Tyr-172 and Glu-174. In general, binding of PPACK seems to stabilize several side chains (Leu-95d, Glu-97, Glu-174, and Glu-218) that are disordered in the KLK2-BEN structure.

The Pro-217–Glu-218–Pro-219 motif (Fig. 3*C*), which is unique among the human kallikreins, most likely rigidifies the entrance frame of the S1 pocket. Notably, it also tilts the backbone carbonyl group of Gly-216 by at least 30° compared with typical serine protease geometries, resulting in an orientation of Gly-216-O that requires sufficient backbone flexibility of the P2 residue to allow a favorable hydrogen bond geometry between Gly-216-O and P3-N. The conformation of Gly-216 explains why specificity profiling disallows proline in P2 (Fig. 2*D*); P2-Pro will induce a P3 main chain geometry that impedes the antiparallel β-sheet interactions between the backbone of the P3 residue and Gly-216, which serves as S3 residue in KLK2.

In the KLK2-BEN structure, a second benzamidine molecule (BEN 302) is located at the lower back of KLK2. Its amidine group forms hydrogen bonds to Trp-20-Nϵ1, Glu-23-N, Tyr-137-Oη, and two water molecules from a symmetry-related protein molecule. To check whether this secondary benzamidine binding site only existed in this interface, we scored the interactions between KLK2 and BEN 302 with DSX. This program evaluates protein-ligand complexes by using a knowledge-based scoring function where increasingly negative scores indicate more favorable interactions. If DSX considered both symmetry-related molecules, it reported a score of −62.3. This value is comparable to the score of benzamidine bound to the S1 site (−65.4) and less negative than the score of the strongly binding ligand PPACK (−130.9). However, if DSX neglected contributions of the symmetry-related molecule, the score of BEN 302 increased to −26.6. Thus, the secondary benzamidine binding site presumably requires formation of the crystal lattice, whereas it is absent in solution.

##### Loops around the Active Site Cleft

**FIGURE 5.**
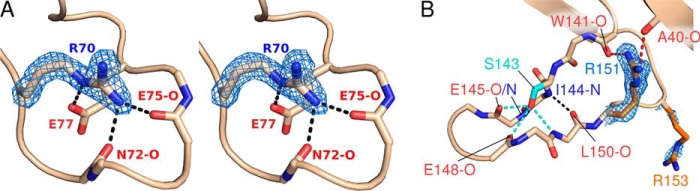
**KLK2 surface loops.** *A*, stereo view of the 75-loop. The guanidinium group of Arg-70 interacts with putative calcium ligands, thereby precluding calcium binding. Hydrogen bonds are shown as *black*, *dashed lines. B*, the 148-loop is stabilized by hydrogen bonds that involve Ser-143 (*cyan*, *dashed lines*), Arg-151 (*red*, *dashed lines*), or backbone atoms only (*black*, *dashed line*). Maximum-likelihood 2*mF*_obs_ − *DF*_calc_ electron density contoured at 1.5σ is shown in *blue* for the side chains of arginines 70, 151, and 153.

Although calcium ions are well known allosteric modulators of trypsin (85) or factor IXa, they did not stimulate the proteolytic activity of KLK2 (data not shown). The 75-loop of trypsin provides the Glu-70 and Glu-80 side chains as well as the Asn-72 and Val-75 carbonyl-O atoms as Ca^2+^ ligands with two water molecules, completing the octahedral calcium binding site (86), which is comparable to the site found in factor IXa (87). By contrast, the 75-loop of KLK2 exhibits Arg-70 and Gly-80, respectively (Fig. 5*A*). Furthermore, the guanidinium group of Arg-70 essentially replaces the Ca^2+^ by formations of hydrogen bonds to Glu-77-Oϵ1, Asn-72-O and Glu-75-O. This arrangement is also comparable to the 75-loop of human thrombin (79), which contains a Lys-70.

The autolysis or 148-loop forms the basement of the active site cleft and is highly similar in the classical KLKs. In KLK2 the 148-loop assumes a slender L shape that is mostly stabilized by hydrogen bonds between Ile-144-N and Leu-150-O as well as those of Ser-143-Oγ with Glu-145-N, Glu-145-O, Glu-148-O, and Leu-150-N (Fig. 5*B*). Arginines 151 and 153 of KLK2 are unique among the kallikreins (see Fig. 3*C*). The side chain of Arg-151 confers extra stability to the loop by contacting both Trp-141-O and Ala-40-O. In contrast, the more flexible and ill-defined side chain of Arg-153 protrudes into the solvent, which may explain why proteolysis after Arg-153, but not after Arg-151 has been observed (88, 89).

**FIGURE 6.**
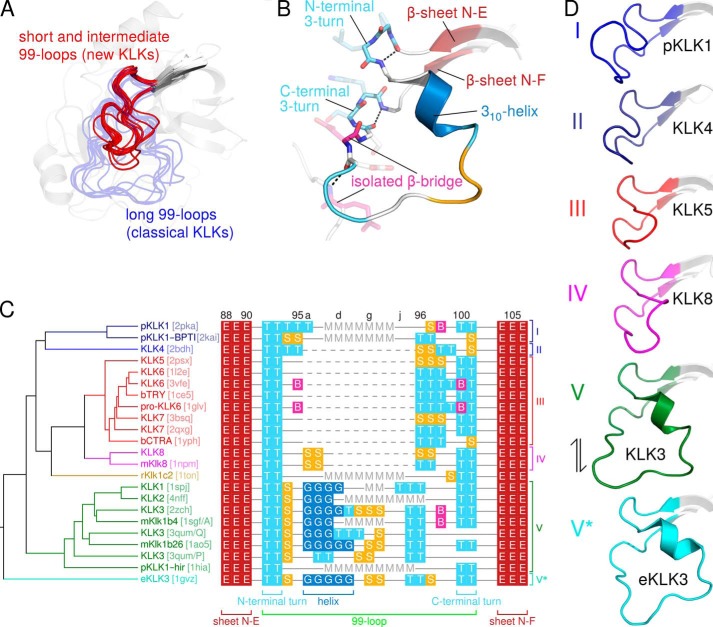
**99-loops of the KLK family.** *A*, an alignment of all KLK 99-loops whose structures are known generally classifies them as either short/intermediate (*red*) or long (*blue*, *semitransparent*). *B*, secondary structure of the long KLK3 99-loop, colored according to *C. C*, in the structure-based alignment of 99-loop sequences (*right*), each residue is labeled by the DSSP single-letter code for its secondary structure (instead of the single-letter amino acid code): *B*, isolated β-bridge; *E*, extended β-sheet; *G*, 3_10_-helix; *S*, bend; *T*, turn. In addition, *M* denotes missing residues, *dashed lines* indicate gaps, and *continuous lines* represent residues that lack secondary structure. Sequence numbers appear on *top*, and *brackets below the alignment* label conserved secondary structure elements. After structural superposition, backbone Cα distances, *i.e.* pairwise r.m.s.d. values, for the corresponding 99-loop stretches were calculated with a script in the molecular graphics program PyMOL (supplemental File S2), resulting in a distance matrix. The UPGMA dendrogram (*left*) is based upon pairwise 99-loop Cα r.m.s.d. and organizes the 99-loops in six clusters, which are indicated to the *right of the alignment*. PDB codes are given in *brackets*. The dendrogram was calculated by PHYLIP (64) and drawn by TreeGraph 2 (72). *D*, representative members of the six 99-loop clusters I-V and V*. The *double-headed arrow* indicates that cluster V and V* loops probably represent interconvertible open and closed conformations.

As pointed out above, residues of the 99- or kallikrein loop contribute to nonprimed substrate binding sites. Because this loop greatly varies in length among the members of the KLK family, it is a distinguishing structural feature. To compare the 99-loop of KLK2 with other 99-loops whose conformation is already known, we aligned all available KLK structures: human KLKs 1–8, porcine KLK1 (pKLK1), rat tonin (rKlk1c2, formerly called rat kallikrein 2), equine KLK3 (eKLK3), mouse kallikreins (mKlks) 1b4, 1b26 (formerly called mouse glandular kallikrein 13), and 8 as well as bovine trypsin and chymotrypsin A. As evident from Fig. 6*A*, 99-loops may be generally classified as short, intermediate, or long. Short 99-loops lack any insertion with respect to chymotrypsin (KLKs 4–7, 14, and 15). Intermediate 99-loops contain 2–8 additional amino acids after residue 95 (KLKs 8–13, mKlk8). Long 99-loops with 11 additional amino acids (residues 95a to 95k) characterize the so-called classical kallikreins KLK1–3, pKLK1, rKlk1c2, mKlk1b4, mKlk1b26, and eKLK3. In the respective crystal structures most of the long 99-loops lack electron density for at least two central residues. Likewise, the KLK2-BEN and KLK2-PPACK structures lack any interpretable electron density for Leu-95d to Glu-97 and Lys-95e to Asp-96, respectively. Apparently, the notable insertion confers extra flexibility to this loop. Nevertheless, the 99-loop is entirely well defined in mKlk1b26, KLK3, and eKLK3. In these structures the kallikrein loop is rigidified by interactions with a symmetry-related molecule (eKLK3), with another molecule in a noncrystallographic dimer (mKlk1b26), or with a stabilizing antibody and *N*-linked glycans (KLK3).

An exhaustive analysis of their secondary structure (Fig. 6, *B* and *C*) revealed that all 99-loops contain two conserved hydrogen-bonded 3-turns, one at their N terminus (91-O to 94-N) and one at their C terminus (99-O to 102-N). The latter presumably stabilizes the catalytically efficient orientation of Asp-102. Furthermore, all long 99-loops that are sufficiently well resolved contain a short 3_10_-helix, which starts at residue 95a. However, we sought to extend these rather qualitative observations by a quantitative approach. To this end we performed a multiple alignment of KLK structures from which we calculated a symmetrical distance matrix. Matrix elements represented the pairwise 99-loop dissimilarities, *i.e.* r.m.s.d. between equivalent 99-loop Cα atoms. Using this distance matrix, hierarchical clustering based on the UPGMA algorithm yielded a 99-loop dendrogram (Fig. 6*C*). Accordingly, the 99-loops belong to six structurally similar types or clusters (Fig. 6*D*). Cluster I comprises the long 99-loops of free pKLK1 (PDB ID 2pka) and of pKLK1 in complex with bovine pancreatic trypsin inhibitor (PDB ID 2kai); cluster II only contains the short 99-loop of KLK4 (PDB ID 2bdh). Although the pKLK1 and KLK4 99-loops differ in length, they both bend away from the active site and form a roof above the substrate binding site. All other 99-loops (clusters III to V*) bend toward the active site cleft or even form a lid over it. Loops in cluster III (KLK5 (PDB ID 2psx), KLK6 (PDB IDs 1l2e and 3vfe), pro-KLK6 (PDB ID 1gvl), and KLK7 (PDB IDs 2qxg and 3bsq) resemble the short loops of trypsin (PDB ID 1ce5) and chymotrypsin (PDB ID 1yph). The 99-loops of human KLK8 and mouse Klk8 (PDB ID 1npm) in cluster IV exhibit a comparable conformation, but a three-residue elongation induces an additional bend at its midpoint. Cluster V comprises the long 99-loops of human KLKs 1 (PDB ID 1spj), 2 (PDB IDs 4nfe and 4nff), and 3 (PDB ID 2zch and 3qum/chains P and Q), pKLK1 in complex with hirustasin (PDB ID 1hia), mKlk1b26 (PDB ID, 1ao5), and mKlk1b4 (PDB ID 1sgf/chain A). Finally, cluster V* only contains the kallikrein loop of eKLK3 (PDB ID 1gvz). Type V and V* loops represent interconvertible open and closed conformations, respectively. (24). The active site cleft of human KLK3 (type V) is accessible for a substrate, whereas the 99-, 148-, and 220-loop block the substrate binding site in eKLK3 (type V*) (the nomenclature of clusters V and V* emphasizes the connection between open and closed 99-loop conformations; it also refers to the E-E* equilibrium of conformational selection, which is discussed below). The central eight residues of the rKlk1c2 99-loop (PDB ID 1ton) are disordered, and the remainder of the loop seems markedly distorted, as two of its residues participate in Zn^2+^ binding. As a consequence, we did not assign this loop any cluster.

##### Irreversible Inhibition by 99-Loop Autolysis

The close proximity of the 99-loop to the active site cleft prompted us to investigate its influence on the inactivation of KLK2 by autolysis and on its inhibition by divalent zinc cations. Mature KLK2 underwent autolysis in solution at a single site within the 99-loop (Lys-95e↓His-95f), which was confirmed by N-terminal sequencing. To confirm that autolysis depended on Lys at position 95e, we mutated this residue to Met or Gln. Indeed, the KLK2 mutants K95eM and K95eQ were nearly as active as the wild type (Table 2) but completely resisted autolytic 99-loop cleavage. Besides, they displayed less unspecific cuts during EK-mediated activation, most likely because EK was unable to cleave after Met95e or Gln95e and due to a more compact conformation of intact KLK2. Interestingly, Lys-95e resides next to the single putative *N*-glycosylation site of KLK2 at Asn-95 (90). Glycosylation at the latter residue might limit access to the cleavage site, which would explain why 99-loop cleavage has never been reported for KLK2 isolated from natural sources.

**TABLE 2:** **Kinetic constants of KLK2 variants and IC_50_/*K_i_* values for their inhibition by Zn^2+^ ions** Proteolytic activity was measured in 100 μl of assay buffer (50 mm Tris-HCl, pH 7.5, 100 mm NaCl, 10% (v/v) DMSO, 0.1% (w/v) BSA) containing 400 ng (150 nm) of KLK2 and 250 μm chromogenic substrate (Bz-Pro-Phe-Arg-pNA unless otherwise indicated). *k*_cat_ values were normalized according to active site titration. The error of the catalytic efficiency *k*_cat_/*K_m_* was calculated according to the Fenner (61) formula from the respective standard errors. ND, not determined. Bz-PFR, Bz-Pro-Phe-Arg-pNA; H-GHR, H-Gly-His-Arg-AMC; H-PFR, H-Pro-Phe-Arg-pNA.

KLK2/mutant	*K_m_*	*k*_cat_	*k*_cat_/*K_m_*	IC_50_	*K_i_**^a^*
	μ*m*	*s*^−*1*^	*m*^−*1*^ *s*^−*1*^	μ*m*	μ*m*
WT (Bz-PFR)	75 ± 2	7.94 ± 0.13	105,900 ± 3,300	22 ± 1	4.9 ± 0.4
WT (H-GHR)	146 ± 7	ND	ND	19 ± 3	7 ± 1
WT (H-PFR)	69 ± 3	23.42 ± 0.19	339,400 ± 15,000	ND	ND
H25A	85 ± 6	ND	ND	23 ± 6	6 ± 2
H91A	115 ± 18	ND	ND	6 ± 1	2 ± 1
K95eM	68 ± 4	4.10 ± 0.11	60,300 ± 3,900	15 ± 1	3.1 ± 0.4
K95eQ	107 ± 20	7.46 ± 0.23	69,700 ± 13,200	35 ± 4	11 ± 3
H95fA	68 ± 7	ND	ND	60 ± 8	13 ± 3
H101A	233 ± 16	2.27 ± 0.12	9,700 ± 800	13 ± 1	6 ± 1

*^a^* The *K_i_* value was calculated assuming competitive inhibition, *K_i_* = IC_50_/(1 + [S]/*K_m_*), where the substrate concentration [S] was 250 μm (27).

**FIGURE 7.**
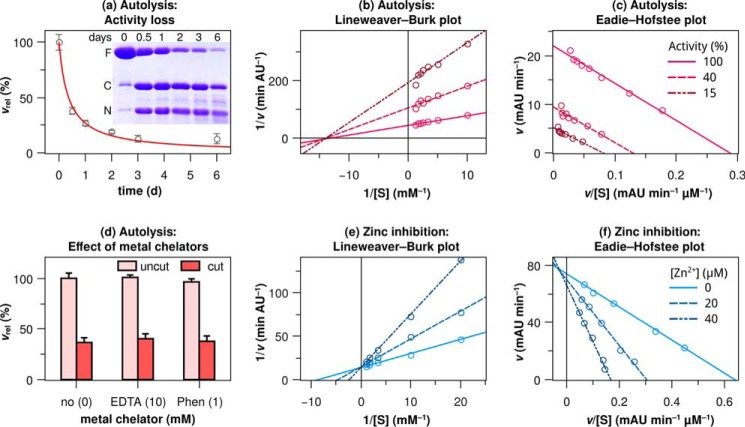
**KLK2 inactivation and inhibition kinetics.** *a*, time-dependent activity loss points toward a second-order reaction mechanism. The regression curve illustrates the corresponding rate law, *d*[KLK2]/d*t* = −2 *k* [KLK2]^2^, with [KLK2]_0_ = 100% and *k* = 0.014 ± 0.002 d^−1^, resulting in a time dependence of 1/(2*kt* + 1). *Inset*, time-dependent 99-loop cleavage as evident from nonreducing SDS-PAGE. *F*, full-length KLK2; *C*, C-terminal fragment (His-95f–Pro-245a); *N*, N-terminal fragment (Ile-16–Lys-95e). Shown are Lineweaver-Burk (*b*) and Eadie-Hofstee plot (*c*) of autolytic inactivation. Substrate concentration-dependent reaction velocities were measured at three time points during autolysis when 100, 40, and 15% of KLK2 were active, respectively. These plots confirm that autolysis inactivates the protease in a way that is analogous to noncompetitive inhibition, as the regression lines intersect at the *x* axis (*b*) or run in parallel (*c*). *AU*, absorbance units. *d*, Zn^2+^ chelators did not restore proteolytic activity of KLK2 with a cut 99-loop (*Phen* is the Zn^2+^-specific chelator 1,10-phenanthroline-5-amine). Lineweaver-Burk (*e*) and Eadie-Hofstee (*f*) plots indicate that inhibition of KLK2 by Zn^2+^ is competitive, as the regression lines almost intersect at the *y* axis in both plots. All measurements involved 150 nm wild type KLK2 and 250 μm Bz-PFR-pNA. Relative velocities *v*_rel_ are relative to the highest velocity in the respective panel.

Intriguingly, autolysis had a major impact on proteolytic activity; KLK2 with a cleaved 99-loop was completely inactive toward proteinaceous (itself) and chromogenic substrates (Bz-PFR-pNA). The time dependence of activity loss coincided with progressive 99-loop cleavage (Fig. 7*A* and its *inset*) and indicated a second-order reaction mechanism, which agrees well with autolysis in *trans*, *i.e.* KLK2 + KLK2 → KLK2 + cut KLK2. Also, the mutants K95eM and K95eQ became gradually inactive during storage at room temperature over several days (not shown). Contrary to wild type KLK2, the slow activity loss of these mutants corresponded to a decrease in their concentration instead of 99-loop cleavage. Apparently, even the substitution of a single amino acid destabilized KLK2 and facilitated its precipitation, which is in line with a noticeable reduction in refolding yields for all KLK2 mutants. We further addressed possible mechanisms for the unexpected enzyme inactivation triggered by the 99-loop cleavage. Obvious possibilities are (i) an increase in affinity for Zn^2+^, which itself inhibits KLK2 activity, (ii) blockage of the substrate recognition sites (*K_m_*-type inhibition), or (iii) disruption of the catalytic triad (*k*_cat_-type inhibition).

We could exclude option (i), which is 99-loop autolysis did not inactivate KLK2 by increasing its affinity for Zn^2+^; neither 10 mm EDTA nor 1 mm 1,10-phenanthroline-5-amine was able to reactivate clipped KLK2 (Fig. 7*D*). Because the 99-loop defines the S2 and S4 sites, but not the S1 site, we expected that 99-loop-cleaved KLK2 would still bind to immobilized benzamidine. The observed binding affinity exceeded that of wild type KLK2, indicating the accessibility of the S1 site with possible subtle changes in the binding geometry. Next, we tested if cleaved KLK2 would still react with H-Arg-AMC and the active site titrant 4-nitrophenyl-4-guanidinobenzoate, which require only an intact S1 site for binding. Importantly, this was not the case. Together, these findings would speak against option (ii) and rather for option (iii), *i.e. k*_cat_-type inhibition. Thus, 99-loop cleavage primarily affects the catalytic triad and possibly the presentation of the bound P1 residue to Ser-195. Indeed, 99-loop cleavage inactivates KLK2 in a way that is analogous to noncompetitive inhibition (Fig. 7, *B* and *C*). To further investigate the degree of distortion of the catalytic residues, we employed PPACK as a competitor to benzamidine binding. PPACK harbors an extraordinarily electrophilic carbonyl carbon, compensating for a partially disrupted triad. Indeed, both intact and cleaved KLK2 were able to react with PPACK at a 10-fold molar excess (*i.e.* 100 μm PPACK added to 8 μm KLK2), as evidenced by the lack of binding to a benzamidine-coupled resin. These observations support the notion of a distorted, but not completely disrupted, catalytic triad and oxyanion pocket. Proteolysis within the 99-loop may perturb the triad in the following way; cleavage between Lys-95e and His-95f generates two novel, flexible termini. Movement of these termini dislocates Asp-102 and concomitantly distorts the catalytic triad.

##### Reversible Inhibition by Zinc

Zn^2+^ ions inhibit wild type KLK2 in the micromolar range (Table 2). After incubation with 200 μm Zn^2+^ for 16 h followed by the addition of 10 mm EDTA, 99% of the initial activity was recovered. Lineweaver-Burk and Eadie-Hofstee plots illustrate that the inhibition type is almost perfectly competitive (Fig. 7, *E* and *F*). KLK2 shares this susceptibility to Zn^2+^ with the prostatic KLKs 3 and 4 and with the epidermal KLKs 5, 7, and 14 (91). In both tissues high Zn^2+^ concentrations presumably modulate KLK activity (92, 93). The lack of structural evidence for a Zn^2+^ binding site prompted us to investigate Zn^2+^ inhibition by a panel of KLK2 mutants (Table 2). In each mutant we substituted alanine for one residue, which contributes to known inhibitory Zn^2+^ sites in other KLKs. Substitutions of residues Lys-95e and His-95f reduced the inhibitory effect not >3-fold, which suggests that other residues are the critical Zn^2+^ ligands.

## DISCUSSION

Substrate specificity as determined by positional scanning somewhat disagrees with specificity inferred from phage display (94). These discrepancies may result from the different proteases that were used in these experiments. Although we carried out positional scanning with KLK2 expressed in prokaryotes, phage display employed KLK2 from human samples, which most likely was glycosylated. Furthermore, subsite cooperativity may influence the phage display results. Investigation of cooperativity between subsites requires other methods, such as enzyme kinetic measurements of synthetic substrates with systematically varying residues in one position. Despite a Lys:Arg ratio of about 1:2 in positional scanning, phage display-derived peptides contained P1-Arg in 40 of 41 cases. A similar, almost exclusive occurrence of Arg in P1 has been reported for physiological KLK2 substrates (14) and for complexes of KLK2 with proteinaceous inhibitors (95, 96). The atypical acceptance of Pro in P1, but not in P2, hints toward a kinked substrate binding mode preceding the scissile peptide bond. In phage display, aromatic side chains only appeared four times (twice in P2 and once in P1′ and P4′) but never in the remaining positions. Instead, small or uncharged residues were preferred in P3, P2, P1′, and P2′ (65, 55, 60, and 70% of the recovered peptides, respectively) followed by hydrophobic residues. Interestingly, P1-Arg and P1′-Ser surrounded the scissile bond in one third of all cases. These findings agree well with the reactive center loop sequence of PCI (P4-FTFR↓SAR-P3′), a highly potent KLK2 inhibitor (*k*_ass_ = 2 × 10^5^ m^−1^ s^−1^) (13). PCI contains P1-Arg and aromatic side chains in P2 and P4 as well as the Arg↓Ser scissile bond. However, KLK2 also forms complexes with serpins whose reactive center loop sequences do not reflect the results from specificity profiling; reactivity toward plasminogen activator inhibitor 1 (P4-VSAR↓MAP-P3′) (20), protease inhibitor 6 (P4-MMMR↓CAR-P3′) (97), and even α_1_-antichymotrypsin (P4-ITLL↓SAL-P3′) (98) demonstrates that substrate recognition depends on exosite properties aside from the active site recognition sequence.

So far, three Zn^2+^ inhibition mechanisms have been described for KLKs. First, Zn^2+^ binding to Glu-77 and His-25 of KLK4 disturbs the Ile-16–Asp-194 salt bridge, which destabilizes the active site (46). Second, Zn^2+^ binding to His-91, His-101, and His-233 of KLK3 has been proposed to pull Asp-102 away from the catalytic triad (99). Such a binding mode has been confirmed in the equine KLK3 homologue (56). Third, Zn^2+^ binding to residues in the 99-loop (*e.g.* His-97 and His-99 in rKlk1c2, His-96 and His-99 in KLK5, Thr-96-O and His-99 in KLK7), and to the catalytic His-57 destroys the active site (47, 51, 55).

**FIGURE 8.**
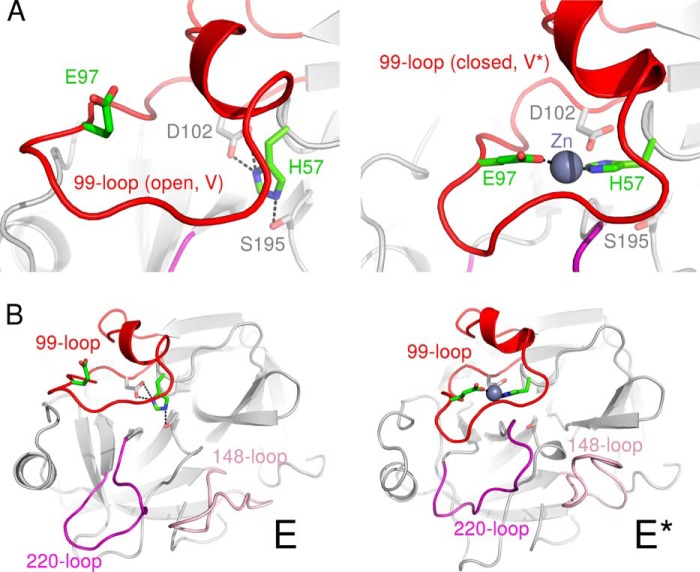
A model of the Zn^2+^-induced E-E* transition in KLK2. Closeup of the 99-loop (*A*) and overall view of KLK2 (*B*) in the active E form (*left*) and Zn^2+^-inhibited E* form (*right*). According to this model Zn^2+^ binding to His-57 and Glu-97 reversibly inactivates KLK2 in two ways. First, Zn^2+^ disrupts the catalytic triad by dislocating His-57. In addition, the 99-, 148-, and 220-loop assume a conformation that occludes the substrate binding site. We obtained a model of the E form by grafting the well resolved 99-loop of KLK3 (PDB ID 2zch) onto the KLK2-PPACK structure. The coordinates of eKLK3 (PDB ID 1gvz) provided the basis for the model of the E* form. Additionally, we mutated Asp-97 to Glu and complemented the Zn^2+^ binding site by choosing an appropriate rotamer of His-57.

Essentially unaltered Zn^2+^ inhibition of the KLK2 mutants H25A, H91A, and H101A (Table 2) allowed us to exclude a KLK4- or KLK3-like Zn^2+^ inhibition mechanism for KLK2. Instead, we believe that Zn^2+^ binds to His-57 and at least one residue in the C-terminal region of the 99-loop. This region contains three potential Zn^2+^ ligands (Asp-96, Glu-97, and Asp-98). Asp and Glu side chains represent 15 and 12% of all reported Zn^2+^ ligands (His, 38%; Cys, 29%) (100). In the KLK2-PPACK structure with its open 99-loop conformation (type V), the side chains of Glu-97 and His-57 are separated by about 12 Å. However, a counterclockwise rotational movement of the 99-loop would yield a closed, eKLK3-loop conformation (type V*) and might also create a Zn^2+^ binding site by moving Glu-97 closer to His-57 (Fig. 8*A*). Such a rearrangement would be based on the observed plasticity of the 99-loop, as corroborated by its highly flexible region that lacks electron density in both KLK2 structures. By contrast, only residues 95g and h are disordered in KLK1, which indicates that its 99-loop is relatively rigid and might explain why Zn^2+^ ions do not inhibit KLK1. *N*-Glycosylation at both Asn-95 and Asn-95f and *O-*glycosylation at Ser-95b (101) may additionally decrease the flexibility of the KLK1 99-loop. Subtle variations in the *K_i_* value of KLK2 mutants (Table 2) provide further evidence for a Zn^2+^-induced 99-loop shift. The H91A and K95eM mutations exhibit a decreased *K_i_* value that may correspond to an increase in 99-loop flexibility. Conversely, mutants with an increased *K_i_* value (K95eQ, H95fA) may contain a more rigid 99-loop. Notably, the *K_i_* value of the H25A mutant (residue 25 is far away from the 99-loop) is almost equal to the one of wild type KLK2.

**FIGURE 9.**
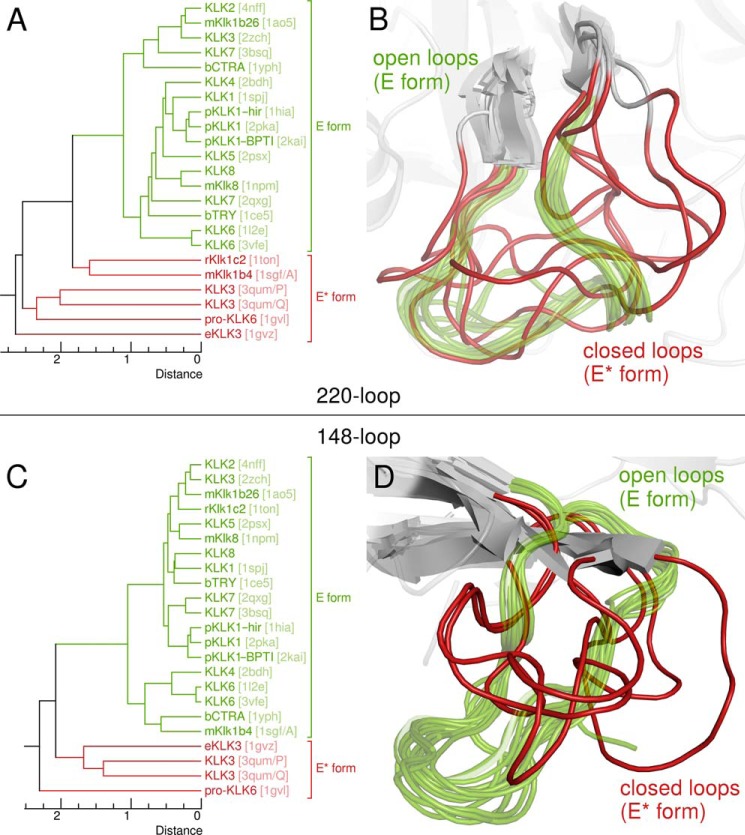
**Open and closed 220- and 148-loop conformations.** *A* and *C*, UPGMA dendrograms derived from pairwise 220- or 148-loop Cα r.m.s.d., which were calculated after structural superposition as for the 99-loop with a PyMOL script (Fig. 6, supplemental File S2); PDB codes are given in *brackets. B* and *D*, alignments of all KLK 220- or 148-loops whose structures are known. According to these superpositions, both loops may assume open (*green*, *semitransparent*) or closed (*red*) conformations, which correspond to the E and E* form of the protease, respectively.

If Zn^2+^ binding alters the conformation of the KLK2 99-loop as described above, it may also promote further structural changes that inactivate the protein. Several KLK structures (KLK3 (PDB ID 3qum/chains P and Q), pro-KLK6, rKlk1c2, eKLK3, and mKlk1b4) have been proposed to represent such inactive conformations. When we compared KLK2 to eKLK3 (56), we observed several prominent changes. On the one hand, eKLK3 lacks an oxyanion hole due to a 170° change of the dihedral angle ϕ between Gly-193-N and -Cα (KLK2, 100°; eKLK3, −70°). Similarly, the oxyanion hole is absent in pro-KLK6 and KLK3 (PDB ID 3qum/chains P and Q) as these structures miss the activating salt bridge between the N terminus and Asp-194 (25, 50). On the other hand, three loops next to the active site cleft change their conformation, namely the 99-, 148-, and 220-loop. The latter loop is notable for its role in the conformational selection mechanism exhibited by trypsin-like proteases; it exists in two different conformations that either occlude the active site (known as the E* form) or render it accessible to a substrate (the E form) (102, 103). As we confirmed by visual inspection and calculation of UPGMA dendrograms based on Cα r.m.s.d. (Fig. 9), not only the 220-loop but also the 148-loop assumes the E* form in all KLK structures that are presumably inactive.

These observations suggest a compelling possibility for Zn^2+^ ions to inactivate KLK2 by modulating its E-E* equilibrium. According to this model, structural changes of the 99-, 148-, and 220-loop accompany the E-E* transition (Fig. 8*B* and supplemental Movie S3). An open (type V) 99-loop stabilizes the E form of the 220-loop. By contrast, Zn^2+^ binding promotes the closed (type V*) 99-loop conformation, which favors the E* form of the 220-loop. Indeed, Arg-95g approaches Glu-218 in an inactive KLK3 structure (PDB ID 3qum/chain Q) (25). Together with the movement of the 148-loop, these relocations block the substrate binding site. Existence of a Zn^2+^-induced E* form that comprises a closed 99-loop conformation agrees with several experimental results that are otherwise hard to explain. First, occlusion of the active site cleft upon Zn^2+^ binding accounts for the apparently competitive inhibition by Zn^2+^ (Fig. 7*E*, F) even if the displacement of His-57 rather suggests a noncompetitive inhibition mechanism. Conformational selection implies that KLK2 has to assume its E form before it can bind to a substrate. This transition expels the Zn^2+^ from the 99-loop and simultaneously restores the catalytic triad. Second, we may explain why Zn^2+^ remarkably increases the affinity of KLK2 for benzamidine-coupled resin. Both Zn^2+^-free and Zn^2+^-inhibited KLK2 bind to this resin during chromatography. However, although benzamidine concentrations beyond 25 mm elute Zn^2+^-free KLK2, Zn^2+^-inhibited protein remains bound even in the presence of 100 mm benzamidine is due to a strongly reduced *k*_off_. By overlapping the substrate binding site, the 99-loop and the spacer arm that couples benzamidine to the resin completely block access to the S1 site for benzamidine in the elution buffer. Third, it becomes clear why soaking with Zn^2+^ ions invariably destroyed KLK2 crystals grown in the absence of Zn^2+^; the concomitant rearrangement of the 99-, 148-, and 220-loop severed the crystal contacts that involve these loops.

## CONCLUSIONS

In summary, we were able to complement the structures of the classical KLKs 1 and 3 by solving the structure of KLK2. Although both KLK2 structures represent Zn^2+^-free forms of the protease, mutational analysis located a Zn^2+^ binding site in the 99-loop. Intriguingly, this loop also contains an autolysis site, whose cleavage inactivated KLK2. Hence, our novel structural and enzymatic data are in line with earlier findings, which have established KLK2 as a key player of the semen liquefaction cascade. Accordingly, male fertility demands that at least four factors tightly regulate KLK2, namely its N-terminal propeptide, inhibition by Zn^2+^ ions, permanent inactivation by cleavage of surface loops, and complex formation with PCI. Whereas the 99-loop limits KLK2 to certain substrates by accommodating their P2 and P4 residues, it may also confine proteolytic activity to a narrow post-ejaculatory time frame; this loop contains Zn^2+^ binding residues for reversible inhibition and might even conduct the concerted movements of other surface loops (148-, 220-loop), which mark the transition between active and inactive states of trypsin-like proteases. Thus, we consider the 99-loop as master regulator of KLK2 activity and possibly in related KLKs.

## Supplementary Material

Supplemental Data

## Footnotes

3 S. Moore, unpublished data.

4 M. Debela, V. Magdolen, W. Skala, C. S. Craik, E. Schneider, W. Bode, H. Brandstetter, and P. Goettig, manuscript in preparation.

5 A. Razeto, B. Galunsky, V. Kasche, K. S. Wilson, and V. S. Lamzin, unpublished data.

## Footnotes

2 The abbreviations used are:

KLK: kallikrein-related peptidase
AMC: 7-amino-4-methylcoumarin
BENAC: benzamidine (BEN) affinity chromatography
EK: enterokinase
IEC: ion exchange chromatography
PCI: proteinase C inhibitor
pNA: *para*-nitroaniline
Bz: benzoyl
PPACK: H-D-Phe-Pro-Arg chloromethyl ketone
r.m.s.d.: root mean square deviation
UPGMA: unweighted pair group method with arithmetic mean.
