# Supplementary material for: Structure-Function Analyses of Human Kallikrein-related Peptidase 2 Establish the 99-Loop as Master Regulator of Activity
Source: J Biol Chem. 2014 Oct 17;289(49):34267–83. doi: 10.1074/jbc.M114.598201 (PMC4256358; doi:10.1074/jbc.M114.598201)
Supplement: Supplemental Data [file supp_289_49_34267__index.html]

Structure-Function Analyses of Human Kallikrein-Related Peptidase 2 Establish the 99-Loop as Master Regulator of Activity — Structure-Function Analyses of Human Kallikrein-related Peptidase 2 Establish the 99-Loop as Master Regulator of Activity — Structure of Human Kallikrein-related Peptidase 2 — Supplemental Data 

# Structure-Function Analyses of Human Kallikrein-related Peptidase 2 Establish the 99-Loop as Master Regulator of Activity

## Supplemental Data

**Files in this Data Supplement:**

- Supplemental file 1 (.txt, 30 KB) - CIF library for the ligand PPACK and its covalent bonds to His57 and Ser195
- Supplemental file 2 (.txt, 1 KB) - PyMOL script for the calculation of RMSD distances between equivalent C&#x26;#945;?atoms of surface loops
- Supplemental file 3 (.avi, 6.3 MB) - Movie of the conformational changes in a model of the Zn2+-induced E?E\* transition in KLK2
- Supplemental file 4 (.docx, 17 KB) - Supplement legends with reference
