# Supplementary material for: Structure-Function Analyses of Human Kallikrein-related Peptidase 2 Establish the 99-Loop as Master Regulator of Activity
Source: J Biol Chem. 2014 Oct 17;289(49):34267–83. doi: 10.1074/jbc.M114.598201 (PMC4256358; doi:10.1074/jbc.M114.598201)
Supplement: Supplemental Data [file supp_M114.598201_jbc.M114.598201-4.docx]

# SUPPLEMENTARY FILE LEGENDS

**SUPPLEMENTARY FILE S1.** *Source code of HIS-PPACK-Ser.lib.* This CIF library describes the ligand PPACK (0G6) and its two covalent bonds to His57 and Ser195.

**SUPPLEMENTARY FILE S2.** *Source code of ca_rmsd_matrix.py.* The PyMOL command ca_rmsd_matrix calculates the current RMSD between equivalent Cα atoms in a selection (i.e., Cα atoms in different objects but with equal residue numbers). The command considers all objects and writes the resulting distance matrix to a PHYLIP infile.

**SUPPLEMENTARY MOVIE S3.** *A model of the Zn^2+^-induced E–E* transition in KLK2.* This movie illustrates the conformational changes described in Figure 8. In addition, the active site cleft of the E form binds the octapeptide P4-ILSR↓IVGG-P4′ (orange), which resembles the KLK2 cleavage site in pro-KLK3. Intermediates in the E–E* transition were calculated by the Morph2 server (1).

**SUPPLEMENTARY REFERENCES**

1. Krebs, W. G., and Gerstein, M. (2000) The morph server: a standardized system for analyzing and visualizing macromolecular motions in a database framework. *Nucleic Acids Res.* **28**, 1665–1
